# Light Higgs channel of the resonant decay of magnon condensate in superfluid ^3^He-B

**DOI:** 10.1038/ncomms10294

**Published:** 2016-01-08

**Authors:** V. V. Zavjalov, S. Autti, V. B. Eltsov, P. J. Heikkinen, G. E. Volovik

**Affiliations:** 1 Department of Applied Physics, Low Temperature Laboratory, Aalto University, PO Box 15100, FI-00076 AALTO Espoo, Finland; 2 Landau Institute for Theoretical Physics, acad. Semyonov av., 1a, 142432, Chernogolovka, Russia

## Abstract

In superfluids the order parameter, which describes spontaneous symmetry breaking, is an analogue of the Higgs field in the Standard Model of particle physics. Oscillations of the field amplitude are massive Higgs bosons, while oscillations of the orientation are massless Nambu-Goldstone bosons. The 125 GeV Higgs boson, discovered at Large Hadron Collider, is light compared with electroweak energy scale. Here, we show that such light Higgs exists in superfluid ^3^He-B, where one of three Nambu-Goldstone spin-wave modes acquires small mass due to the spin–orbit interaction. Other modes become optical and acoustic magnons. We observe parametric decay of Bose-Einstein condensate of optical magnons to light Higgs modes and decay of optical to acoustic magnons. Formation of a light Higgs from a Nambu-Goldstone mode observed in ^3^He-B opens a possibility that such scenario can be realized in other systems, where violation of some hidden symmetry is possible, including the Standard Model.

## Abstract (web summary)

The order parameter describing the spontaneous symmetry breaking which occurs when a system becomes a superfluid is analogous to the Higgs field in particle physics from which the Higgs boson arises. Here, the authors demonstrate the existence of a light Higgs boson in the B-phase of superfluid ^3^He.

## Graphical abstract

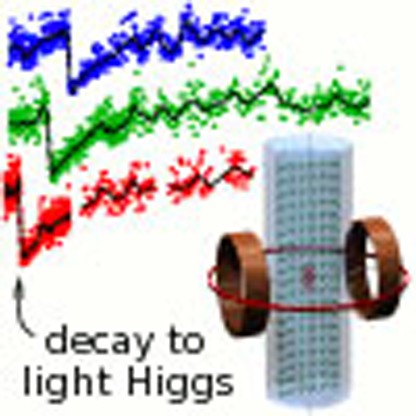

The superfluid transition in ^3^He, a fermionic isotope of helium, occurs due to formation of Cooper pairs with orbital momentum *L*=1 and spin *S*=1. The corresponding order parameter is a 3 × 3 matrix of complex numbers, which includes both spin and orbital degrees of freedom^1^. Thus, besides fermionic quasiparticles, superfluid ^3^He possesses 18 bosonic degrees of freedom, collective modes (oscillations) of the order parameter. Each mode has a relativistic spectrum 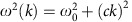, with a mode-specific wave velocity *c* and a gap (or mass) *ω*_0_. Modes with non-zero *ω*_0_ are Higgs modes and others are Nambu-Goldstone (NG) modes. Each NG mode corresponds to spontaneously broken continuous symmetry of the normal state.

In conventional superconductors with the order parameter of a single complex number, only the symmetry with respect to the change of the wave-function phase is broken. This leads to one NG phase mode and one-amplitude Higgs mode, which was experimentally observed^2^,^3^,^4^.

In unconventional B-phase of superfluid ^3^He, the symmetry with respect to relative rotations of the spin and orbital spaces is additionally broken^5^, and the order parameter in the zero magnetic field is


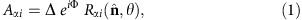


where Δ is the gap in the fermionic spectrum, Φ is the phase and *R*_*αi*_ is a rotation matrix, which connects spin and orbital degrees of freedom. The matrix *R*_*αi*_ is represented in terms of the rotation axis 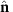 and angle *θ*. Parameters Φ, 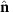 and *θ* determine a four-dimensional subspace of degenerate states. Thus, among 18 collective modes of ^3^He-B (Fig. 1a), four are NG modes: oscillation of Φ is sound and oscillations of *R*_*αi*_ (or 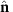 and *θ*) are spin waves. The other 14 modes are the Higgs modes with energy gaps of the order of Δ. These heavy Higgs modes have been investigated for a long time both theoretically^6^,^7^,^8^,^9^ and experimentally^10^,^11^,^12^,^13^,^14^.

In superconductors and in the Standard Model, the NG bosons become massive due to the Anderson-Higgs mechanism^15^,^16^,^17^. In electrically neutral ^3^He, these modes are gapless, when viewed from the scale of Δ∼100 MHz (set by the critical temperature *T*_c_∼10^−3^ K). At low-energy scale of ∼1 MHz, corresponding to the frequency of our NMR experiments, two weak effects become significant: spin–orbit interaction and applied magnetic field **H** (Fig. 1b).

Spin–orbit interaction lifts the degeneracy with respect to *θ*, and the minimum energy corresponds to the so-called Leggett angle *θ*_L_=arccos(−1/4). This explicit violation of the symmetry of the B-phase leads to appearance of the gap for the *θ* mode. This mode becomes an additional, light, Higgs boson. The gap value *Ω*_B_ is called Leggett frequency, it is a measure of the spin–orbit interaction. At low temperatures 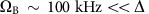.

Two other spin-wave modes are oscillations of 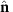. In the magnetic field, the equilibrium state corresponds to 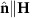 and the field splits these two modes in the same way as in ferromagnets. One of the modes, the optical magnon, acquires the gap equal to the Larmor frequency *ω*_L_=*γH* (where *γ* is the gyromagnetic ratio). Another one, the acoustic magnon, remains gapless, but its spectrum becomes quadratic. Such unusual form of the spectrum comes from a violation of the time-reversal symmetry by the magnetic field (for general discussion of the NG modes with quadratic spectrum see ref. 18).

All three low-frequency spin-wave modes are described by the closed system of Leggett equations^1^. In particle physics, such set of low-energy modes, which includes NG modes and a light Higgs, is called the Little Higgs field^19^. Formal definition of such field in ^3^He-B is given in Supplementary Note 1.

In an NMR experiment, one follows the dynamics of magnetization **M**, or of the spin **S** of the sample. The motion of *θ*, corresponds to longitudinal spin waves, 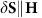, while oscillations of 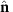 correspond to transverse spin motion, *δ***S**||**H**. Optical magnons can be directly created with traditional transverse NMR. With a suitable coil system, one can also directly excite longitudinal spin oscillations, or light Higgs mode^20^. Coupling to short-wavelength acoustic magnons is hard to achieve in a traditional NMR experiment with large excitation coils.

In this work, we use a technique, based on Bose-Einstein condensate (BEC) of optical magnons^21^, to probe interaction and conversion between all components of the little Higgs field in ^3^He-B. As a result, we observe parametric decay of optical magnons to light Higgs bosons, and both parametric and direct conversion between optical and acoustic magnons. The measured mass of light Higgs and propagation velocity of acoustic magnons are close to the expected values. Thus, we experimentally confirm the little Higgs scenario in ^3^He-B. The little Higgs field appears in quantum chromodynamics^22^, where NG modes (pions) acquire light mass due to the explicit violation of the chiral symmetry, which is negligible at high energy, but becomes significant at low energy^23^. The relatively small mass of the 125 GeV Higgs boson observed at the Large Hadron Collider suggests that it might be also the pseudo-Goldstone (light Higgs) boson (see for example, ref. 24 and references therein).

## Results

### Suhl instability

As a tool to study dynamics of the little Higgs field in superfluid ^3^He-B, we use trapped BECs of optical magnons (Fig. 2). The condensate is well separated from the container walls, where the strongest magnetic relaxation in ^3^He usually occurs^25^. Thus, tiny relaxation effects, connected to coupling of optical magnons to other components of the little Higgs field, can be observed.

**Figure 1:**
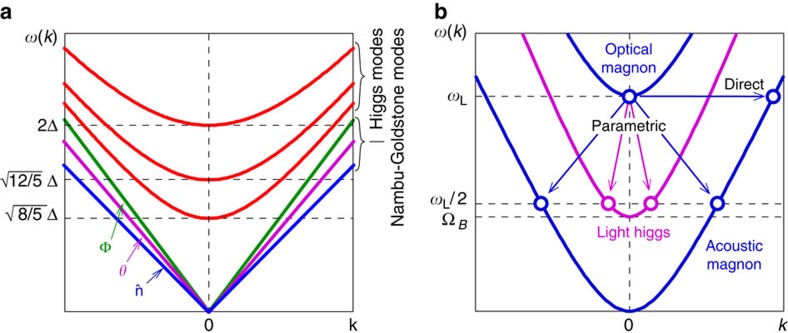
Illustration of collective-mode spectra in ^3^He-B. (**a**) Modes at high-energy scale (*ω*∼Δ∼100 MHz). From top to bottom there are six separate branches. Three red lines show heavy Higgs modes: four degenerate pair-breaking modes with the gap 2Δ, five imaginary squashing modes with the gap 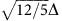 and five real squashing modes with the gap 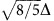. Nambu-Goldstone modes include a sound mode (propagating oscillations of Φ), a spin-wave mode that corresponds to propagating oscillations of *θ*, two spin-wave modes that correspond to propagating oscillations of 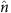. These modes are gapless at the high-energy scale and have different propagation velocities *c*. (**b**) Spin-wave modes at low-energy scale (*ω*∼10^−3^Δ∼100 kHz). Because of spin–orbit interaction, the *θ* mode acquires a small gap 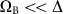 and becomes a light Higgs boson. Two 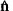 modes are split by the magnetic field into optical and acoustic magnons. Arrows indicate decay channels observed in our experiments.

**Figure 2:**
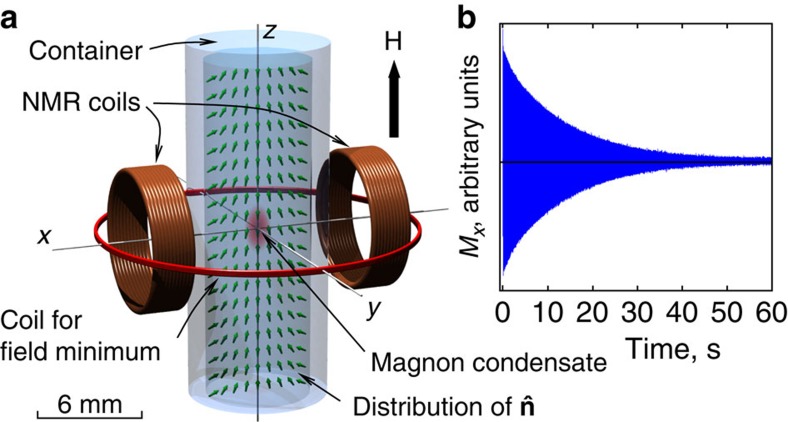
Experimental setup. (**a**) Superfluid ^3^He-B is confined in a cylindrical quartz container, and a constant magnetic field **H** is applied along the container axis. A special coil creates a minimum of the field magnitude *H* in the axial direction, while transverse NMR coils are used to pump optical magnons and to detect magnetization precession. Green arrows show equilibrium distribution of the 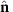 vector, which together with the **H** profile creates a trap for optical magnons near the axis of the sample. In this trap, magnon BEC is formed. (**b**) An example of the signal from the NMR coil during the condensate decay measured at *ω*/2*π*=833 kHz and *P*=0 bar. Its amplitude is proportional to the coherently precessing transverse magnetization of the condensate.

**Figure 3:**
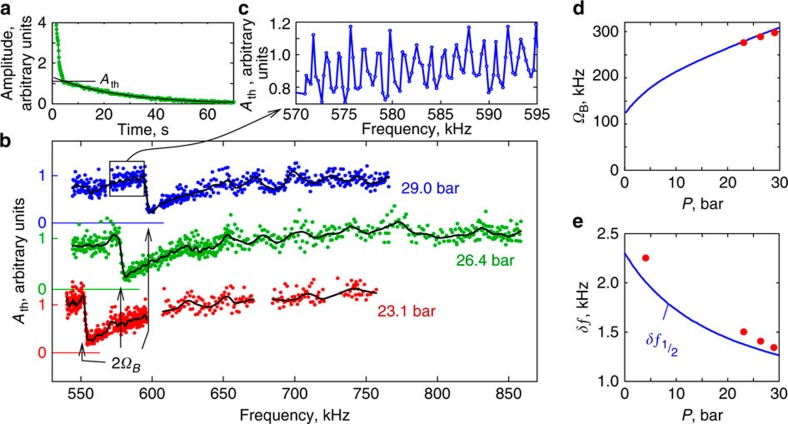
Measurements of the Suhl instability. (**a**) An example measurement of the NMR signal amplitude during the magnon condensate decay obtained at *ω*_L_/2*π*=741 kHz and *P*=26.4 bar. A Suhl instability threshold at the amplitude *A*_th_ is clearly seen. (**b**) Dependence of the threshold amplitude *A*_th_ on the frequency of optical magnons *ω*_opt_≈*ω*_L_ (coloured symbols) for three pressures *P*. Sharp decrease is seen at *ω*_opt_=2*Ω*_B_(*P*), where the decay of an optical magnon to two light Higgs bosons becomes possible. Black lines are smoothed data using running average. Curves for different pressures are shifted in the vertical direction and respective zero levels are marked by horizontal lines. (**c**) Zoom to one measurement in (**b**) shows periodic modulation, which corresponds to resonances of acoustic magnons in the experimental container. (**d**) Pressure dependence of the light Higgs boson mass *Ω*_B_ (symbols) from measurements in (**b**). (**e**) Frequency separation of acoustic magnon resonances (symbols) from measurements like in (**c**). Solid lines in (**d**,**e**) are theoretical values based on known ^3^He parameters^28^ without fitting.

When number of pumped magnons is low, slow exponential relaxation of the precession signal is determined by spin diffusion and energy losses in the NMR pick-up circuit^26^ (Fig. 2b). We have found that above some threshold amplitude, the relaxation becomes much faster (Fig. 3a). The explanation is the Suhl instability^27^, a well-known nonlinear effect in magnets when a uniform precession of magnetization (here at the optical magnon frequency *ω*_opt_) parametrically excites a pair of acoustic magnons with twice smaller frequency *ω*_ac_ and opposite **k**-vectors: *ω*_opt_=*ω*_ac_(**k**)+*ω*_ac_(−**k**). In the case of ^3^He-B, both acoustic magnons and the light Higgs modes can be parametrically excited (Fig. 1b). The process occurs with conservation of energy and momentum. The threshold amplitude is inversely proportional to the coupling between decaying and excited waves, and proportional to the relaxation in the excited wave.

### Mass of light Higgs

The measured threshold amplitude as a function of NMR frequency and pressure is plotted in Fig. 3b. The frequency dependence allows us to identify the decay channels. It is clear from Fig. 1b that the decay of the optical magnon to a pair of light Higgs bosons with the frequency *ω*_Higgs_, *ω*_opt_=*ω*_Higgs_(**k**)+*ω*_Higgs_(−**k**), is possible only when the precession frequency is larger then 2*Ω*_B_. We see a pronounced drop of the threshold amplitude at this frequency: the threshold decreases by about an order of magnitude.

In Fig. 3d, the measured mass of light Higgs *Ω*_B_ is plotted as a function of pressure. Measurements are in a good agreement with values of the Leggett frequency from ref. 28.

### Resonances of acoustic magnons

In addition to the sharp drop, connected with light Higgs mode, we find periodic modulation of the threshold amplitude as a function of the frequency of the precession (Fig. 3c). These periodic peaks originate from the parametric decay of the optical magnons in the BEC to acoustic magnons. The frequency dependence is explained by quantization of the magnon spectrum in the cylindrical container, which serves as a resonator for acoustic magnons. Consider a decay of the optical magnon with frequency *ω*_opt_ into acoustic magnons with frequency *ω*_ac_=*Nω*_opt_, where for the parametric excitation *N*=1/2. In the following discussion, we will use *ω*_opt_=*ω*_L_, since the difference is negligible for trapped optical magnons. By sweeping the magnetic field, we can change both magnon spectrum and magnon trap and observe resonances in the cell. The simple resonance condition for acoustic magnons in a cylinder with the radius *R* gives the distance between the resonances (Supplementary Note 2):


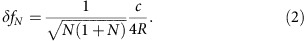


where *c* is the relevant spin-wave velocity.

In Fig. 3e, the measured acoustic magnon resonance period 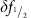 is plotted as a function of pressure. The results are in a good agreement with equation (2), where values of the spin-wave velocity are taken from our recent measurements^29^.

### Effect of quantized vortices

**Figure 4:**
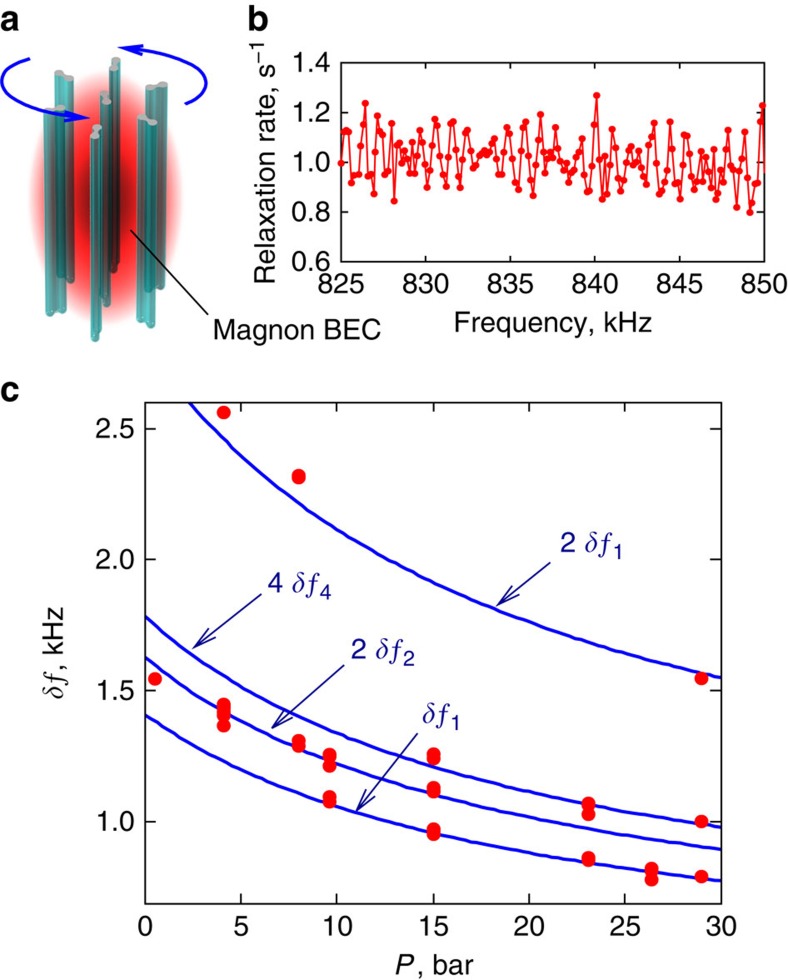
Excitation of acoustic magnons by vortices. (**a**) Schematic plot of vortices in rotating ^3^He-B. Precession of magnetization of the magnon condensate causes oscillations of non-axisymmetric vortex cores. The oscillations produce acoustic magnons and increase relaxation of the condensate. (**b**) Relaxation rate of the magnon condensate as a function of frequency, measured at *Ω*=1 rad s^−1^ and *P*=23.4 bar. Two sets of acoustic magnon resonances with periods ∼1 kHz produce a clearly seen beat with a period of 5 kHz. (**c**) Measured periods of magnon condensate relaxation peaks (symbols) as a function of pressure. Lines are plotted using equation (2) for acoustic magnon resonances without fitting parameters.

An additional relaxation mechanism for the magnon condensate is found when quantized vortices are formed in the sample. In the presence of these localized topological objects, the momentum **k** of the spin-wave modes is not conserved, and one expects direct excitation of acoustic magnons by the optical mode. We can rotate the sample with angular velocities up to *Ω*=21 rad s^−1^ to create a cluster of rectilinear quantized vortices, which cross the whole experimental region including the magnon BEC (Fig. 4a). In this state, the relaxation rate, plotted as a function of the frequency in Fig. 4b, reveals several periodic sets of peaks. We attribute these peaks to resonances of acoustic magnons with frequencies *ω*_L_, 2*ω*_L_ and so on.

In ^3^He-B, the rotational symmetry of a vortex is spontaneously broken and the vortex core can be treated as a bound state of two half-quantum vortices, which can rotate around the vortex axis. Dynamics of the vortex is affected by the precessing magnetization^30^,^31^. Precession of **S** and 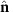 in the magnon BEC produces torsional oscillations of the vortex core. The fact that the equilibrium position of 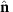 deviates from the vertical direction within the magnon BEC makes these oscillations unharmonic. As a result, acoustic magnons with frequencies *Nω*_L_ can be emitted.

The amplitudes of the various resonances depend on a distribution of vortex cores and the wave nodes of acoustic magnons. For example, an axially symmetric distribution of vortices can excite only symmetric waves, which means doubling of the observed resonance period. In our experiment, acoustic magnons (with wave length 5–10 μm) are emitted by vortices, which are within the magnon condensate. The distance between vortices 0.1–0.2 mm, is comparable with the size of the trapped condensate 0.2–0.4 mm. Thus, the amplitudes of resonances are sensitive to details, such as order-parameter texture, rotation and pressure, and we do not see all the harmonics at all pressures. Nevertheless, the resonance periods plotted in Fig. 4c follow the theoretical values (2) or their multiples (denoted as 2*δf*_1_ and so on).

## Discussion

To summarize, we have observed the interplay of all three spin-wave modes, which form a little Higgs field in superfluid ^3^He-B. In particular, we have found two channels of parametric decay of optical magnons: to a pair of light Higgs bosons and to a pair of acoustic magnons. Although the search for similar resonant production of pairs of Standard Model Higgs bosons reported by the ATLAS collaboration^32^ has not succeeded yet, our results support the basic physical idea behind this effort. Another system where the light Higgs mode can be observed is the multicomponent condensate in cold gases^33^, where interaction between components can be set up to produce the hidden symmetry.

We find that the low-energy physics in superfluid ^3^He has many common features of the Higgs scenario in Standard Model: both are described by the SU(2) and U(1) symmetry groups; the acoustic and optical magnons correspond to the doublet of W^+^ and W^−^ gauge bosons, which spectrum also splits in magnetic field^34^; the light Higgs mode has parallel with the 125-GeV Higgs boson. However, in addition, the ^3^He-B has the high-energy sector with 14 heavy Higgs modes. This suggests that in the same manner, the 125-GeV Higgs boson belongs to the low-energy sector of particle physics, and if so, one may expect the existence of the heavy Higgs bosons at TeV scale.

We have demonstrated that the short-wavelength acoustic magnons can be emitted and detected with the BEC of optical magnons. Acoustic magnons can be lensed by non-uniform magnetic fields and the order-parameter texture, and thus might serve in future as a powerful local probe to study topological superfluidity of ^3^He, including Majorana fermions on the boundaries of the superfluid and in the cores of quantized vortices.

## Methods

### The sample geometry and NMR setup

Superfluid ^3^He-B is placed in a cylindrical container with the inner diameter of 2*R*=5.85 mm, made from fused quartz (Fig. 2). The container has closed top end and open bottom end, which provides the thermal contact to the nuclear demagnetization refrigerator. Static magnetic field is applied parallel to the container axis. A special coil creates a controlled minimum of the field magnitude along the axial direction. Transverse NMR coils, made from copper wire, are used to create and detect magnetization precession. Coils are part of a tuned tank circuit with the *Q* value of ∼130. Frequency tuning is provided by a switchable capacitance bank, installed at the mixing chamber of the dilution refrigerator. To improve signal-to-noise ratio, we use a cold preamplifier, thermalized to liquid helium bath.

The measurements are performed at low temperatures *T*<0.2 *T*_c_, where spin-wave velocities and the Leggett frequency are temperature-independent. Typically, we use *T*=130–350 μK, depending on pressure. The temperature is measured by a quartz tuning fork thermometer, installed at the bottom of the sample cylinder. The heat leak to the sample was measured in earlier work to be ∼12 pW (ref. 35). The measurements are performed at pressures 0–29 bar and in magnetic fields *H*=17–26 mT with corresponding NMR frequencies *ω*_L_/2*π*=550–830 kHz.

### Magnon trap

Minimum of the axial magnetic field forms a trapping potential for optical magnon quasiparticles in the axial direction. Trapping in the radial direction is provided by the spin–orbit interaction via the equilibrium distribution of the order parameter. In this geometry, it forms the so-called flare-out texture: 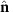 is parallel to **H** on the cell axis and tilted near walls because of boundary conditions^36^. The combined magneto-textural trap is nearly harmonic with trapping length ∼0.3 mm in the radial direction and 1 mm in the axial direction (see Supplementary Note 3 for details).

### Measurements of magnon BEC

Owing to the geometry, the coils couple only to optical magnons with *k*≈0. With a short radio frequncy (rf) pulse in the NMR coils, non-equilibrium optical magnons are created. At temperatures of our experiment the equilibration within the magnon subsystem proceeds much faster that the decay of magnon number, and the pumped magnons are condensed to the ground level of the trap within 0.1 s from the pulse. Manifestation of Bose-Einstein condensation is the spontaneously coherent precession of the condensate magnetization^37^,^38^, which induces current in the NMR coils. The amplified signal is recorded by a digital oscilloscope; an example record is in Fig. 2b. We then perform sliding Fourier transform of the signal with the window 0.3–1 s. In the resulting sharp peak in the spectrum, the frequency determines the BEC precession frequency *ω*_opt_, while the amplitude (such as shown in Fig. 3a) is proportional to the square root of the number of magnons in the trap.

### Rotation

The sample is installed in the rotating nuclear demagnetization refrigerator ROTA^39^, and can be put in rotation together with the cryostat and the measuring equipment. The cryostat is properly balanced and suspended on active vibration isolation, and in rotation the heat leak to the sample remains <20 pW (ref. 35). Vortices are created by increasing angular velocity *Ω* from zero to a target value at temperature ∼0.7 *T*_c_, where the mutual friction allows for fast relaxation of vortex configuration towards an equilibrium array^40^. Further cool-down is performed in rotation.

## Additional information

**How to cite this article:** Zavjalov, V. V. *et al*. Light Higgs channel of the resonant decay of magnon condensate in superfluid ^3^He-B. *Nat. Commun.* 7:10294 doi: 10.1038/ncomms10294 (2016).

## Supplementary Material

Supplementary Information Supplementary Notes 1-3 and Supplementary References
