# Supplementary material for: Light Higgs channel of the resonant decay of magnon condensate in superfluid 3He-B
Source: Nat Commun. 2016 Jan 8;7:10294. doi: 10.1038/ncomms10294 (PMC4729873; doi:10.1038/ncomms10294)
Supplement: Supplementary Information — Supplementary Notes 1-3 and Supplementary References [file ncomms10294-s1.pdf]

SUPPLEMENTARY NOTE 1

Spin waves in ^{3}He-B

Spin waves in^{3}He-B correspond to motions of the rotation matrix R_{aj}. The matrix can be represented by means of the rotation axis $\hat{\mathbf{n}}$ and the rotation angle θ as

$$R_{aj} = \cos\theta\ \delta_{aj} + (1-\cos\theta)\ n_a n_j - \sin\theta\ e_{ajk} n_k. \tag{1}$$

The motion is affected by the energy of the spin-orbit interaction $F_{\rm so}$ and the gradient energy F_∇:

$$F_{\rm so} = \frac{\chi_B \Omega_{\rm B}^2}{15\gamma^2}(R_{jj}R_{kk} + R_{jk}R_{kj}), \tag{2}$$

$$F_\nabla = \frac{1}{2}\Delta^2(K_1G_1 + K_2G_2 + K_3G_3), \tag{3}$$

where

$$\begin{aligned} G_1 &= \nabla_j R_{ak}\nabla_j R_{ak}, \\ G_2 &= \nabla_j R_{ak}\nabla_k R_{aj}, \\ G_3 &= \nabla_j R_{aj}\nabla_k R_{ak}, \end{aligned}$$

χ_B is the spin susceptibility of the ^{3}He-B, γ the gyromagnetic ratio for the ^{3}He atom, $\Omega_{\rm B}$ the Leggett frequency, Δ the superfluid gap, and K_1, K_2 and K_3 are parameters of the gradient energy.

The spin-orbit interaction energy has a simple form in terms of $\hat{\mathbf{n}}$ and θ with a minimum at $\theta = \arccos(-1/4)$:

$$F_{\rm so} = \frac{\chi_B \Omega_{\rm B}^2}{15\gamma^2}\left(\cos\theta + 1/4\right)^2 + {\rm const.} \tag{4}$$

The equation of small spin oscillations near the equilibrium value $\mathbf{S}^0 = (\chi_B/\gamma)\ \mathbf{H}$ is [2]

$$\begin{aligned} \ddot{S}_c &= [\dot{\mathbf{S}} \times \gamma\mathbf{H}]_c \\ &+ \frac{\Delta^2\gamma^2}{\chi_B}\left[K\ \nabla^2\ S_c - K'\ \nabla_j R^0_{cj} R^0_{ak} \nabla_k\ S_a\right] \\ &- \Omega_{\rm B}^2\ \hat{\mathbf{n}}\cdot(\mathbf{S} - \mathbf{S^0})\ \hat{n}_c, \end{aligned} \tag{5}$$

where $K = 2K_1 + K_2 + K_3$ and $K' = K_2 + K_3$.

In a texture with $\hat{\mathbf{n}} \parallel \mathbf{H}$ or in a high magnetic field $\omega_{\rm L}^2/\Omega_{\rm B}^2 \gg 1$ one can separate transverse and longitudinal oscillations. In the case of short wavelengths (when the spin changes on a much shorter distance than the texture) one can write the quasiclassical spectra for plane waves:

$$\begin{aligned} c_\perp^2\ k^2 + (c_\parallel^2 - c_\perp^2)(\mathbf{k}\cdot\hat{\mathbf{l}})^2 + \frac{1}{2}\Omega_{\rm B}^2 \sin^2\beta_n &= \omega(\omega - \omega_{\rm L}), \\ C_\perp^2 k^2 + (C_\parallel^2 - C_\perp^2)(\mathbf{k}\cdot\hat{\mathbf{l}})^2 + \Omega_{\rm B}^2\cos^2\beta_n &= \omega^2, \end{aligned} \tag{6}$$

where β_n is an angle between $\hat{\mathbf{n}}$ and $\mathbf{H}$, the orbital anisotropy axis $\hat{l}_j = R_{aj}S^0_a$ and the spin wave velocities are introduced as

$$\begin{aligned} c_\perp^2 &= \frac{\gamma^2\Delta^2}{\chi_B}(K - K'/2), \quad c_\parallel^2 = \frac{\gamma^2\Delta^2}{\chi_B}K, \\ C_\perp^2 &= \frac{\gamma^2\Delta^2}{\chi_B}K, \quad C_\parallel^2 = \frac{\gamma^2\Delta^2}{\chi_B}(K - K'). \end{aligned} \tag{7}$$

The spin wave velocities are anisotropic, they have different values if the wave propagates in the direction of $\hat{\mathbf{l}}$ or in the perpendicular direction. The second line of Supplementary equation (6) describes a longitudinal wave, the light Higgs mode with a "relativistic" spectrum

$$\omega_{\rm Higgs} = \sqrt{\Omega_{\rm B}^2 + (C\ k)^2}. \tag{8}$$

The first line of Supplementary equation (6) describes two modes of transverse waves, optical and acoustic magnons, with spectra of the form:

$$\omega_{\rm opt} = \frac{\omega_{\rm L}}{2} + \sqrt{\left(\frac{\omega_{\rm L}}{2}\right)^2 + (c\,k)^2}, \quad \omega_{\rm ac} = -\frac{\omega_{\rm L}}{2} + \sqrt{\left(\frac{\omega_{\rm L}}{2}\right)^2 + (c\,k)^2}. \tag{9}$$

In Supplementary equations (8) and (9) the effects of anisotropy and of the spin-orbit interaction are omitted for simplicity.

Little Higgs field for spin waves in ^{3}He-B

Let us introduce a vector field

$$\mathbf{n} = \hat{\mathbf{n}} \sin\theta/2\,. \tag{10}$$

The spin-orbit interaction (4) provides a “Mexican Hat” potential for the **n**-field

$$F_{\rm so} = \Lambda(|\mathbf{n}|^2 - n_0^2)^2\,. \tag{11}$$

where $n_0^2 = 5/8$ and parameter $\Lambda = \frac{32}{15}\frac{\chi_B}{\gamma^2}\Omega_{\rm B}^2$.

In the terminology of particle physics the **n**-vector serves as the “little Higgs” field. In the vacuum states the amplitude of the field is fixed, $|\mathbf{n}| = n_0$, while they are degenerate with respect to the orientation of $\hat{\mathbf{n}}$. The broken $SU(2)$ symmetry leads to two Nambu-Goldstone modes (propagating oscillations of the orientation of $\hat{\mathbf{n}}$), and one light Higgs mode (propagating oscillations of the amplitude $|\mathbf{n}|$ around n_0). These three modes comprising the little Higgs field are similar to the bosonic sector of Standard Model, where also the $SU(2)$ symmetry is instrumental. This low energy sector of Standard Model contains the NG modes (the gauge bosons) and one “light Higgs” (the 125 GeV Higgs boson). Our two Nambu-Goldstone spin-wave modes correspond to the doublet of the W-bosons. The spectrum of the spin wave modes in ^{3}He-B splits in magnetic field into acoustic and optical modes. The similar splitting is discussed for the spectrum of the W-bosons in magnetic field (see e.g. Ref. [3]). Moreover, in strong magnetic fields the Bose condensation of the W-bosons is expected, which is similar to the Bose condensation of optical magnons.

SUPPLEMENTARY NOTE 2

Resonance condition for acoustic magnons

Let’s consider an excitation of acoustic magnons with frequency $N\,\omega_{\rm L}$. Here $N = 1/2$ corresponds to the parametric exitation, $N = 1, 2\ldots$ to the exitation of acoustic magnons with frequency $\omega_{\rm L}, 2\,\omega_{\rm L}, \ldots$. The resonances observed in the experiments correspond to standing waves in the cylindrical cell with radius R. For the short spin waves the resonances can be treated in the quasiclassical approximation:

$$2\int_0^R k_r\;dr = n\pi\,, \tag{12}$$

where k_r is the classical trajectory along the cell diameter and n is integer quantum number. This quantization corresponds to the wave modes in cylinder with high radial and small azimuthal quantum numbers.

The effect of the spin-orbit interaction on the spectrum of short-wave acoustic magnons can be neglected, but anisotropy of wave velocity is important. The ratio of the velocities for $\mathbf{k} \parallel \hat{\mathbf{l}}$ and $\mathbf{k} \perp \hat{\mathbf{l}}$ is $c_\parallel/c_\perp \approx \sqrt{4/3}$. Substituting the transverse magnon spectrum (6) without the spin-orbit term into (12) and taking into account that $\omega = N\omega_{\rm L}$ we get

$$\omega_{\rm L} = \frac{1}{\sqrt{N(1+N)}}\,\frac{\pi n\,c}{2R} \tag{13}$$

where c is a harmonic mean velocity in the non-uniform texture:

$$1/c = \frac{1}{R}\int_0^R \left(c_\perp^2 + (c_\parallel^2 - c_\perp^2)\,\sin^2\beta_l(r)\right)^{-1/2} dr, \tag{14}$$

and β_l is an angle between $\hat{\mathbf{l}}$ and $\mathbf{H}$.

The distance between the resonances is:

$$\delta f_N = \frac{1}{2\pi}\,\frac{\partial \omega_{\rm L}}{\partial n} = \frac{1}{\sqrt{N(1+N)}}\,\frac{c}{4R}\,. \tag{15}$$

Note that the spin wave spectra (6) have been obtained with the assumption of zero coupling between transverse and longitudinal modes ($\omega^2/\Omega_{\rm B}^2 \gg 1$ or $\beta_n \ll 1$). In our experiment this condition is approximately valid for directly excited magnons with $\omega > 2\Omega_{\rm B}$. We use the same approximation also for parametrically excited magnons with $\omega \approx \Omega_{\rm B}$. This is probably the reason why the agreement of the experimental data with Eq. (15) is much better for the directly excited magnons.

SUPPLEMENTARY NOTE 3

Trap for magnon quasiparticles

In the case of optical magnons with $\omega \approx \omega_{\rm L}$, localized in the center of the cell, where $\hat{\mathbf{n}}$ is almost parallel to $\mathbf{H}$, equation (5) can be rewritten in a form of a Schrödinger equation for magnon quasiparticles, where complex value $s_+ = \frac{1}{\sqrt{2}}(S_x + iS_y)$ plays role of the wave function and precession frequency ω plays role of the energy. Effect of texture on the gradient terms is neglected here because it adds only a small correction to the total gradient energy.

$$\left[-\frac{c_\perp^2}{\omega_{\rm L}}\,(\nabla_x^2 + \nabla_y^2) - \frac{c_\parallel^2}{\omega_{\rm L}}\,\nabla_z^2 + \frac{\Omega_B^2}{2\omega_{\rm L}}\sin^2\beta_n + \omega_{\rm L}\right] s_+ = \omega\; s_+ \tag{16}$$

Non-uniform potential for magnons is formed by the order parameter texture and the magnetic field (β_n and $\omega_{\rm L}$ parameters).

$$U = \frac{\Omega_{\rm B}^2}{2\gamma H}\sin^2\beta_n + \omega_{\rm L}. \tag{17}$$

In our setup the potential has a quadratic minimum in the center of the sample: in the flare-out texture angle β_n near the sample axis is linear, $\beta_n = \beta_n' r$ and magnetic field of the longitudinal coil has also quadratic profile near the center.

We use pulsed NMR to populate a few lowest levels in this harmonic trap. If the number of magnons in the system is small enough, interaction between the levels is negligible and the excited states can be resolved in the measurements independently. If the magnon population is above a certain threshold, they collapse to the ground state and form a Bose-Einstein condensate.

From the spectra of the magnon levels in the trap we can find values of spin-wave velocities $c_\perp$ and $c_\parallel$. This work is presented in Ref. 4.

SUPPLEMENTARY REFERENCES

[1] Thuneberg, E.V. Hydrostatic theory of superfluid ^{3}He-B. *J. Low Temp. Phys.* **122**, 657–682 (2001).
[2] Theodorakis, S. & Fetter, A.L. Vortices and NMR in rotating ^{3}He-B. *J. Low Temp. Phys.* **52**, 559–591 (1983).
[3] Chernodub, M.N. Superconducting properties of vacuum in strong magnetic field, *Int. J. Mod. Phys. D* **23**, 1430009 (2014).
[4] Zavjalov, V.V., Autti, S., Eltsov, V.B. & Heikkinen, P.J. Measurements of the anisotropic mass of magnons confined in a harmonic trap in superfluid ^{3}He-B. *JETP Letters* **101**, 802–807 (2015).
